# Control of Excitation of Cladding Modes by Tapering an Insertion of Special Fiber

**DOI:** 10.3390/s21072498

**Published:** 2021-04-03

**Authors:** Diomid D. Bakurov, Oleg V. Ivanov

**Affiliations:** 1 Ulyanovsk Branch of Kotel’nikov Institute of Radio Engineering and Electronics of Russian Academy of Sciences, Ulitsa Goncharova 48, 432071 Ulyanovsk, Russia; ufire@mv.ru; 2 Radio Engineering Faculty, Ulyanovsk State Technical University, Ulitsa S. Venets 32, 432027 Ulyanovsk, Russia

**Keywords:** optical fiber, cladding mode, fiber insertion, mode coupling, taper

## Abstract

A method for controlling the excitation of cladding modes by tapering special fiber insertions made of SM450 and coreless fibers is proposed. The coupling coefficients between the core mode and the cladding modes of the tapered fiber insertion are calculated. For the calculation, changes in the effective refractive indices and phases of the fiber core and in the cladding modes upon tapering are found. The field distribution of the core mode of the standard fiber transmitted through fiber insertion is obtained. The transmission characteristics of insertions of SM450 and coreless fibers during tapering are simulated and compared with the experiment. The possibility of controlling the transmission and excitation of various cladding modes is confirmed experimentally.

## 1. Introduction

Recent interest in the study of optical fiber structures with insertions of special fibers is due to the possibility of exciting cladding modes and using them in fiber-optic sensors [1,2,3]. Such structures can be highly sensitive to environmental parameters such as temperature [4], humidity [5], liquid level [6,7], etc. Some systems with fiber insertions are capable of gas sensing [8] and chemical sensing [9]; for example, they can determine the concentration of ethyl alcohol [10]. The insertion of special fiber can use coreless fiber [11], multi-core fiber [12], taper [10], thin-core fiber [13,14], or multimode fiber [15]. A combination of two insertions can be used as well [16]. Chemical sensing with high performance has also been demonstrated in biosensors using structures based on coated fiber probes with the effect of surface plasmon resonance [17,18,19].

The sensitivity of structures with insertions increases significantly if an interferometer is made, which couples different fiber modes [20,21]. The amplitude of the interference signal depends on the amount of light intensity coupled to the cladding mode by the insertion. The transmission of insertion of a special fiber depends on its parameters: refractive index profile, length, and shape [1]. The length of the insertion should be controlled with high accuracy. If the length of the insertion is insufficient, cladding modes inside the insertion do not have the length to gain the necessary phase difference and the core mode is restored at the insertion output with a low resulting sensitivity of the interferometer. If the insertion length is too large, then the cladding modes come at the end of the insertion with such a phase difference that the amplitude of the core mode may be quite small [2]. Therefore, the insertion length must be selected in such a way that the intensity remaining in the core should be at the level of the amplitude of one of the excited cladding modes, i.e., 20–50% [2]. The required length of the insertion depends on the type of special fiber used and lies in the range from 0.1 mm to a few millimeters.

The production of structures with fiber insertions of these lengths requires a special cleaving procedure with high precision down to micrometers along the fiber length. This presents significant difficulties. In addition, after splicing the structure and measuring its transmission, the insertion length cannot be adjusted to obtain the required values. In this case, it is necessary to create the structure with the insertion again.

In this paper, we propose a method for adjusting mode coupling in the fiber by increasing the length of the insertion heated and stretched it in electric arc discharges. We calculate the length of the insertion as a function of the taper waist radius. The dependence of the taper waist radius on the number of discharges and the discharge time is investigated. We study theoretically and experimentally mode coupling in the insertions of coreless and SM450 fibers with an increase in the insertion length produced by tapering. For this study, we calculate the complete sets of fiber modes, including the core and cladding modes, which are found for decreasing fiber radius. The electromagnetic field at the input end of the insertion is decomposed into the complete set of modes, which propagates to the output end with different phase velocities, where they are summed up. The resulting field is partially coupled to the core mode of the standard fiber, which is measured by a spectrum analyzer. The spectral characteristics of the transmission coefficient for the insertions observed during tapering of the fiber are obtained.

## 2. Fiber Structure

**Figure 1:**
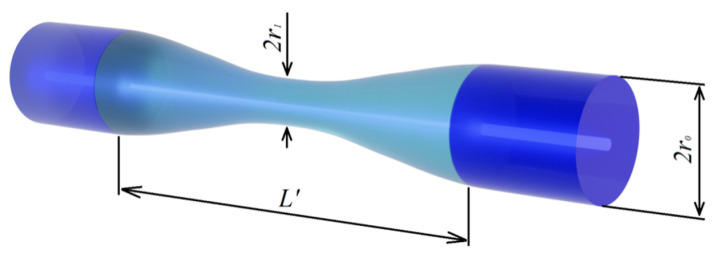
Fiber insertion with taper.

When fiber interferometric structures with insertions are created, special fibers with refractive index profiles that are quite different from that of the standard fiber should be used. We considered a structure consisting of a standard SMF-28 fiber and a piece of special fiber with a length of 0.1–1 mm (Figure 1). In this paper, we investigated two types of fibers for the insertions: coreless fiber with cladding diameter 140 μm and SM450 fiber (manufactured by Fibercore, Southampton, UK) with numerical aperture 0.12, cladding diameter 125 μm, and cutoff wavelength 400 nm. The working range of the SM450 fiber was from 488 to 633 nm. Its core radius was 1.75 µm. As a standard fiber, we used an SMF-28 fiber (manufactured by Corning, Corning, NY, USA) with the following parameters: numerical aperture 0.14, core radius 4.2 μm, cladding radius 62.5 μm, cutoff wavelength 1260 nm, and operating range from 1260 to 1625 nm.

The fibers were spliced using an INNO IFS-10 splicer. A superluminescent diode (manufactured by Superlum, Carrigtwohill, Ireland) was used as a broadband light source, and the spectrum was measured with an Anritsu MS9710 spectral analyzer.

The fabrication of a fiber insertion less than 1 mm in length cannot be carried out in the usual way. To do such short fiber insertions, we developed a special installation based on fiber optic cleaver Fujikura 80s, in which the fiber in the cleaver can be displaced in the longitudinal direction using a micrometer screw. First, the standard fiber was cleaved in a fixed position of the cleaver. After that, a special fiber section was spliced to the standard fiber. Then, the structure was placed in its previous position in the cleaver and was shifted by the required length of 0.1–1 mm, and the special fiber section was cleaved. Another standard fiber was spliced to the resulting structure on the other side in the usual way. In all experiments, the fibers were spliced in a standard operation mode of the splicer.

Despite the fact that the insertion length was controlled in our experiments with an accuracy of about 0.05 mm, this accuracy was insufficient and, therefore, a situation in which the insertion length does not allow us to obtain the required coupling coefficient to the cladding modes is possible. To adjust the length of the insertion, we propose using the arc of a splicing machine heating the insertion. Applying a pulling force to the fiber, the fiber is stretched and a taper is formed in the heated region.

## 3. Tapering the Optical Fiber

To lengthen the insertion, the fiber structure was placed inside the splicer, the fiber was fixed on one side, and a load was suspended on its other side. During discharge, the insertion was heated by an electric arc discharge and was pulled out by the load. Using discharges with duration t=180...220 ms, which is shorter than the time of discharges applied to the fiber during fusion, we were able to increase the insertion length while the cladding diameter was decreased at the same time, and in this way, we could control the coupling coefficient to different cladding modes. The discharge parameters for heating the fiber were the same as in the splicing operation mode but with the time and the number of discharges adjusted.

Let us calculate the change in the shape of the fiber insertion when creating a taper. We assume that the change in the fiber radius is described by the cosine function (this is quite close to the real shape of the taper, as can be seen from the experimental observations) in accordance with the following expression:

(1) $$r(z)=\frac{r_{0}+r_{1}}{2}-\frac{r_{0}-r_{1}}{2}\cos\frac{2\pi z}{L^{\prime }},$$

where $r_{0}$ is the initial radius of the cladding, $r_{1}$ is the radius of the taper waist in the center, and *L’* is the final length of the insertion (Figure 1). The volume of the fiber taper can be found by integrating the taper cross section over fiber length:

(2) $$V=\int_{-L^{\prime }/2}^{L^{\prime }/2}\pi r{\left(z\right)}^{2}dx=\frac{\pi }{8}L^{\prime }\left(3r_{0}^{2}+2r_{0}^{}r_{1}^{}+3r_{1}^{2}\right).$$

Taking into account conservation of the volume of the fiber insertion, it is possible to obtain the following formula for the taper length after stretching:

(3) $$L^{\prime }=\frac{8L{\left(r_{0}/r_{1}\right)}^{2}}{3+2r_{0}/r_{1}+3{\left(r_{0}/r_{1}\right)}^{2}},$$

where *L* is the initial length of the insertion.

**Figure 2:**
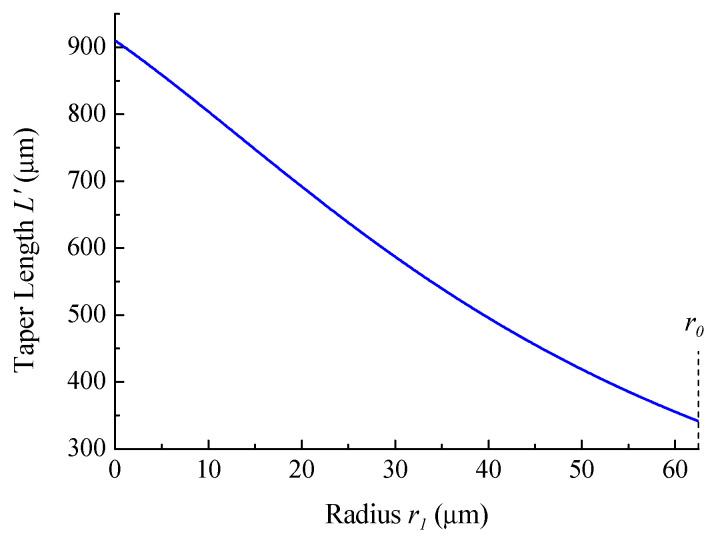
Dependence of taper length on the waist radius for initial taper length L=340 μm.

The dependence of the final taper length on its minimum radius for the initial taper length L=340 μm is shown in Figure 2.

**Figure 3:**
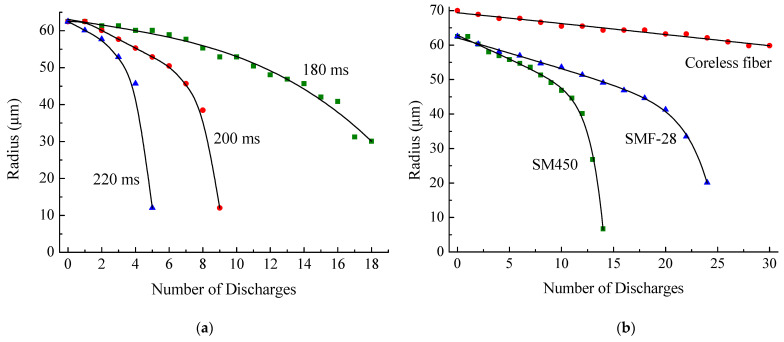
Taper waist radius as a function of the number of discharges (**a**) for the SMF-28 fiber and three values of the discharge time and (**b**) for three fibers and a discharge time of 190 ms.

Using different numbers of electric arc discharges and by varying their time, it is possible to reduce the fiber radius to the required value. At the first stage, it was necessary to find out what time of discharge is suitable for gradual tapering of the fiber. Figure 3a shows the measured dependence of the waist radius of the SMF-28 fiber on the number of discharges for several values of discharge time. The arc power was set equal to the power used during fusion of standard single mode fibers. A tension force of 0.05 N was applied to the fiber. Figure 3b shows the dependence of the waist radius of three different fibers on the number of discharges for discharge time of 190 ms.

It can be seen from the figure that, as the number of discharges increases, the taper formation accelerates. The last discharges greatly change the fiber radius. The optimal discharge times are 180–220 ms. Changing the discharge time by 20 ms almost doubles the rate of taper formation. For small changes in the radius, it is more expedient to use discharges that soften the fiber by a longer exposure because a fewer number of such discharges is needed. However, if the final radius of the taper waist is a small value, long discharges would not work. The dependence of the waist radius for long charge times in the region of small values of radius (45 μm and less) is very steep, and the changes in the radius are too strong even using one discharge. In this case, it is necessary to use short discharges to achieve the desired taper radius continuously. Generally, the experimental results show that the optimal arc time is 190 ms.

**Figure 4:**
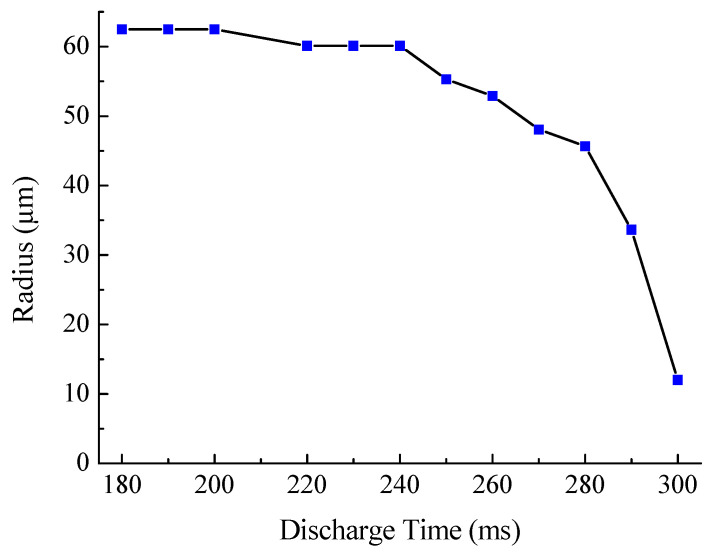
Radius of the taper waist of SMF-28 fiber after one discharge depending on its duration.

When employing tapering for correction of insertion, it is not required to reduce the value of the radius continuously, but the aim is to obtain a certain range of radius values (for example, from 30 to 40 μm); then, a long single discharge can be used. Figure 4 shows the change in the waist radius of the SMF-28 fiber after a single discharge, depending on its time. The obtained dependence is characterized by strong nonlinearity, and when the discharge time is more than 300 ms, the fiber breaks.

The softening temperature of fiber depends on its type (SMF-28, SM-450, or coreless); therefore, before using it as an insertion, it is necessary to perform calibration measurements of the dependence of taper radius on the number and time of discharges for each fiber. As can be seen from Figure 3b, SM-450 requires less discharges to reach the same taper radius; therefore, the softening temperature of SM-450 is lower than that of SMF-28. The initial radius of the coreless fiber is 70 μm, and this fiber is tapered much slower. 

## 4. Calculation of Modes of Optical Fibers

To calculate the transmission of the structure with fiber insertion, the field of the core mode incoming from the SMF-28 fiber at the first junction with the insertion must be decomposed into the modes of the special fiber. In the approximation of adiabatic taper, a set of modes propagates through the insertion without interaction with the second junction, where each mode contributes to the core mode of the standard fiber and they are summed up again. To decompose the mode field of the standard fiber into modes of the special fiber, we found the propagation constants and filed distribution profiles of the modes of these fibers.

When the fiber tapered, the radii of the cladding and core changed; therefore, the tapering changes mode parameters, such as effective refractive index $n_{}^{\left(\mathrm{eff}\right)}(z)=\beta (z)/k_{0}$, where β is the mode propagation constant and $k_{0}$ is the wave number in vacuum. Due to this, each mode gains a different phase after propagation, which leads to a field modification at the insertion end. Therefore, the next stage of this work is to calculate the dependence of the effective refractive indices *n*^(eff)^ on the *z* coordinate.

### 4.1. Corning SMF-28 Fiber

**Figure 5:**
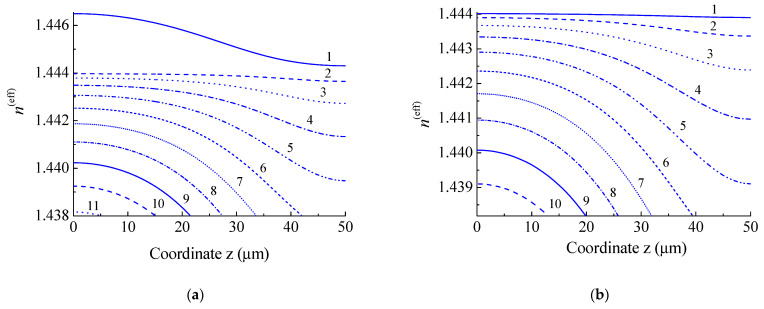
Dependences of effective refractive indices of modes on the z coordinate for (**a**) SMF-28 and (**b**) SM450 fibers at wavelength 1550 nm with radius decreasing from 62.5 to 30.5 μm.

As an example, let us calculate the change in *n*^(eff)^ in the tapered section of a standard SMF-28 fiber, the cladding radius of which is R=62.5 μm, and the radius of the core is 4.2 μm. The refractive indices of the cladding and the core are 1.4440 and 1.4494, respectively. Let the initial length of the insertion be 60 µm; then, the taper length is L=100 µm and the fiber radius in the waist is r=30.7 µm. Effective refractive indices are calculated numerically in the weak guidance approximation by solving Maxwell’s equations in each concentric layer using the method described in [22]. We calculate *n*^(eff)^ from the beginning to the center of the taper. This is sufficient because the taper profile is symmetrical with respect to its center. The dependence of *n*^(eff)^ for the first eleven modes of SMF-28 on the z coordinate is shown in Figure 5a. The curve for LP_01_ mode, which is the core mode, is distinguished from the curves for other modes. When tapering the fiber, the fiber core also shrinks and becomes weaker waveguides; therefore, its effective refractive index approaches the refractive index of cladding mode LP_02_.

### 4.2. Fibercore SM450 Fiber

When creating a fiber interferometer, in particular, an insertion of a special fiber SM450 (manufactured by Fibercore) was used, which has a thin core compared to SMF-28 fiber. The operating wavelength of this fiber is 488–633 nm. The effective propagation constants and mode field profiles were calculated for SM450 fiber with the following parameters: cladding radius—62.5 μm, core radius—$r_{0}=1.75$ μm. The initial length of the insertion is 60 µm. The taper length is L=100 µm, and the fiber radius at the narrowest point is r=30.7 µm. The variation in *n*^(eff)^ for the first ten modes of SM450 fiber depending on the *z* coordinate is shown in Figure 5b. Compared to a standard fiber, the LP_01_ mode of this fiber is a cladding mode; therefore, its effective refractive index is lower than the refractive index of the cladding and the nature of the dependence is the same as that for the other modes.

**Figure 6:**
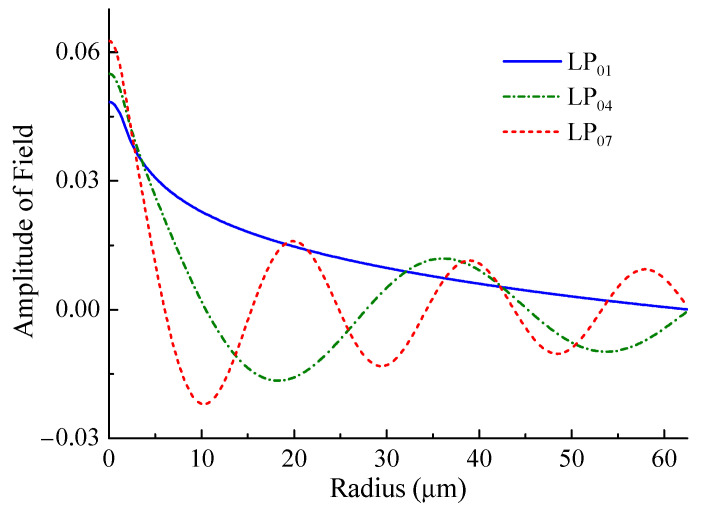
Field distributions of some modes of SM450 fiber at wavelength 1550 nm for cladding radius 62.5 μm.

Using the found values of *n*^(eff)^, one can obtain the distribution of the fields of the fiber modes. Examples of field distributions for some modes are shown in Figure 6. It is seen that the field of the first mode is present in the entire cladding and that its profile is far from the core mode. Therefore, the LP_01_ modes of the standard fiber and SM450 are mismatched and many modes must be excited at their junction.

### 4.3. Coreless Fiber

**Figure 7:**
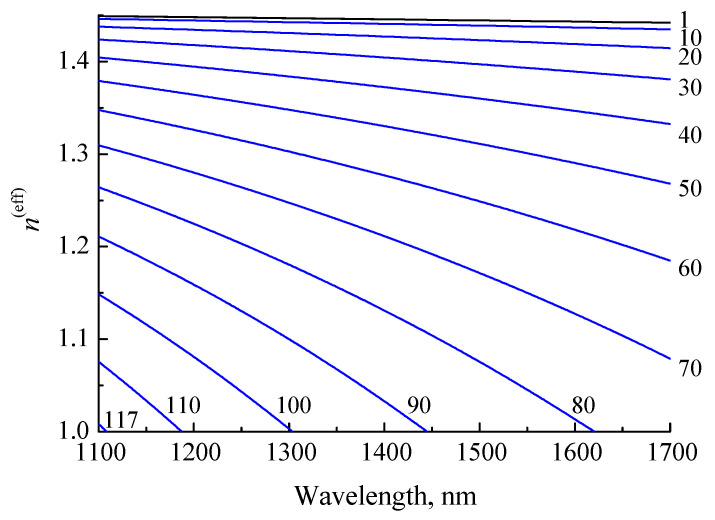
Effective refractive indices of modes of 140 μm coreless fiber versus wavelength. The numbers correspond to the mode number.

Another type of fiber that we used to create the insertion structure was coreless fiber. The light transmitted through such a fiber is not guided by its core. At the beginning of the insertion, the light propagates as in free space, diverging similarly to a Gaussian light beam. If the insertion length is large enough, the beam expands to the outer edge of the fiber and is reflected at this boundary, experiencing total internal reflection. However, calculation of the transmission can be carried out by employing the same method of decomposition of the field into the cladding modes of coreless fiber. When calculating the transmission of light through the insertion, 117 modes of the coreless fiber were found. Their effective refractive indices are shown in Figure 7. The refractive indices decrease monotonically with wavelength and mode number. Since there is no core, all the curves lie below the refractive index of the cladding.

## 5. Simulation of Transmission of Light through Fiber Insertion

The electromagnetic wave enters the insertion from the standard fiber, so we decompose the field of the core mode of SMF-28 fiber into modes of the insertion fiber:

(4) $${E^{\prime }}_{\mathrm{co}}(r)=\sum_{i=1}^{m}c_{i}E_{i},$$

where $E_{i}$ is the field of the *i*th cladding mode of the insertion fiber, ${E^{\prime }}_{\mathrm{co}}$ is the field of the core mode of SMF-28 fiber, and *m* is the number of calculated cladding modes. For this decomposition, we express the coupling coefficients through the following overlap integral [11]:

(5) $$c_{i}=\int E_{i}{E^{\prime }}_{\mathrm{co}}dS=\int_{0}^{\infty }E_{i}(r){E^{\prime }}_{\mathrm{co}}(r)2\pi rdr,$$

**Figure 8:**
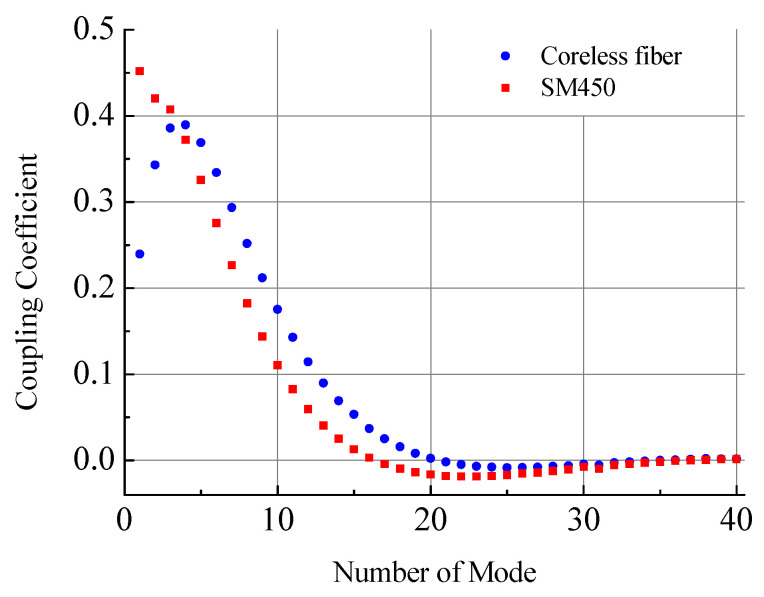
Coupling coefficients for the first 40 modes at the junction between the coreless fiber and SM450 fiber at wavelength 1550 nm.

Here, it is assumed that the amplitudes of the modes are normalized, so each mode carries unit energy flux. The coupling coefficients between the fields of SM450 cladding modes and the field of SMF-28 core mode at the junction of two fibers for the first 40 modes are shown in Figure 8. The energy in each excited mode is determined by the modulus square of the coupling coefficient ${\left|c_{i}\right|}^{2}$. About 90% of energy propagates in the first eight excited modes. The sum of the energies of all modes is 99.968 and 99.987% for SM450 and coreless fibers, respectively. The negligible rest energy is coupled to radiation modes. It can be seen that the maximum coupling efficiency for the coreless fiber is observed for the LP_04_ mode and not for the LP_01_ mode as in the case of SM450.

The theory of fiber tapers has been investigated by many authors, for example, in [23,24,25]. In our further analysis, we assume that the taper formed at the insertion is adiabatic, i.e., the change in its radius is rather smooth and the modes propagate without inter-mode coupling. Then, in order to find the field at the output of the insertion, it is necessary to sum back the modes taking into account the phase change accumulated during propagation through the insertion. The phase change can be calculated using the following formula:

(6) $$\varphi_{i}=k_{0}\int_{0}^{L}n_{i}^{\left(\mathrm{eff}\right)}(z)dz.$$

**Figure 9:**
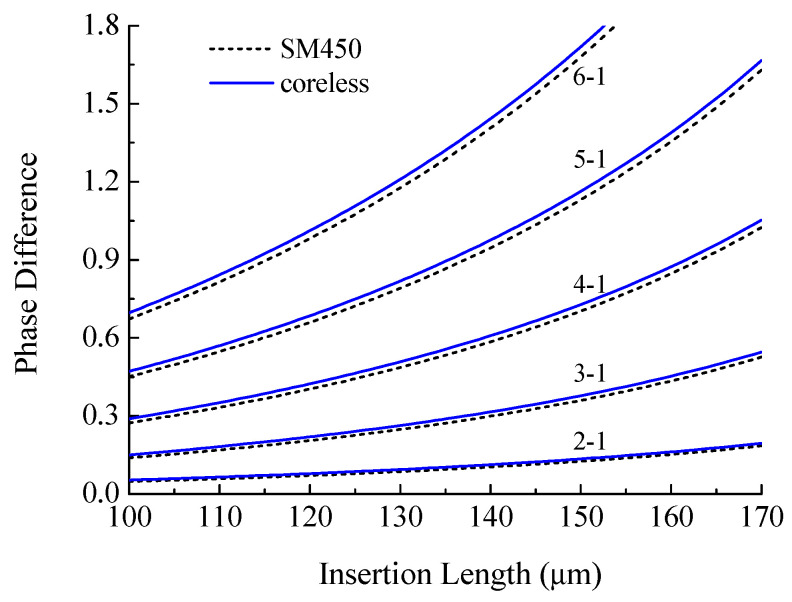
Dependence of the phase incursion difference between the cladding modes and the fundamental mode of SM450 and coreless fibers depending on the taper length.

The phase change was calculated for the case of a decreasing radius of the taper waist from 62.5 to 30.7 μm. The wavelength was taken to be λ=1550 nm. Figure 9 shows the results of calculation of the phase difference between the cladding modes LP_02_–LP_06_ and the fundamental LP_01_ mode. The figure shows the change in the phase difference with elongation of the insertion from 100 to 170 μm. It can be seen that the phase difference increases with lengthening of the taper, and the difference between the modes also increases. The difference between the two types of fiber insertions is not significant.

By adding a phase to the mode amplitudes and summing the fields, we can find the field at the output junction of the structure. Thus, the sum of the fields multiplied by the coupling coefficients gives the field transmitted through the insertion:

(7) $$E_{b}=\sum_{i=1}^{m}c_{i}E_{i}\exp(i\varphi_{i}^{}).$$

**Figure 10:**
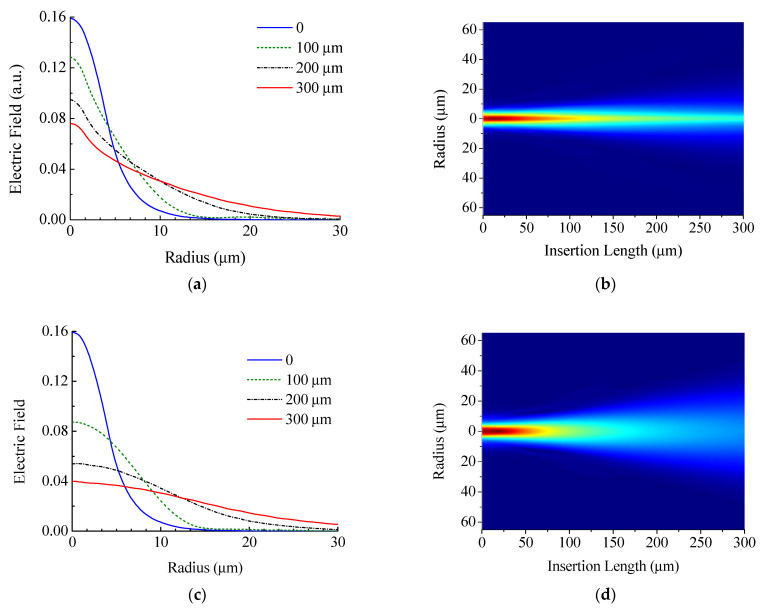
Distribution of the electric field amplitude at different distances in the insertion of (**a**,**b**) SM450 and (**c**,**d**) coreless fibers.

Figure 10 shows the field propagating through the insertion at different positions inside the insertion. As it should be, the field after passing through the zero insertion practically coincides with the field of the main fiber, and as the insertion length increases, the field expands to larger radii.

It can be seen from the figures that the field transmitted through the coreless fiber insertion is wider than that for the SM450 insertion, which can be explained by the presence of a core, although the core of SM450 is a rather weakly guiding waveguide. After propagating half of the insertion length (150 μm) in the fiber without core, the field expands, but it does not practically reach the outer boundary of the fiber, which is located at a radius of 30.7 μm. This fact allows us to consider the propagation of light in a coreless insertion without taking into account the decrease in its radius or a larger radius of the cladding since the physical structure in which the light propagate does not change upon tapering.

At the output junction, the modes of the insertion fiber are coupled back to the SMF-28 core mode. The final transmission coefficient can be obtained through the overlap integral over the fiber cross section between the field in the insertion and the SMF-28 core mode field:

(8) $$T={\left|\int E_{b}{E^{\prime }}_{\mathrm{co}}dS\right|}^{2},$$

**Figure 11:**
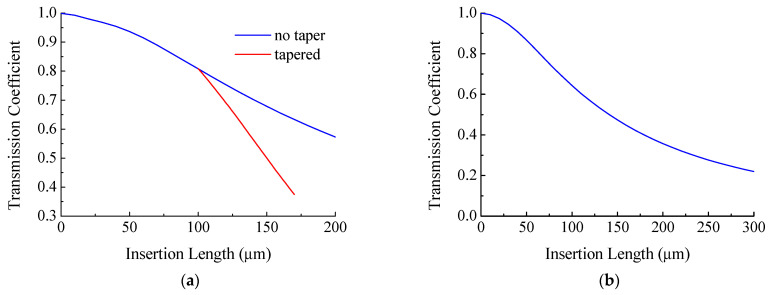
Dependence of the transmission coefficient of insertion with (**a**) SM450 fiber and (**b**) coreless fiber on its length.

Based on the approach described, the transmission coefficient of a fiber structure with SM450 fiber insertion having lengths from 0 to 300 μm was calculated (Figure 11, blue curve). The transmission coefficient decreases monotonically from unity for a fiber without insertion to 0.6 for an insertion of length 200 μm.

The transmission coefficient of the insertion structure was calculated in a similar way when it was tapered (Figure 11, red curve). The initial length of the insertion was L=100 µm, and the radius of the taper waist decreased from R=62.5 µm to r=30.7 µm. The change in taper length was calculated in accordance with Equation (2). It can be seen from the figure that the taper has a lower transmission than the non-tapered insertion.

**Figure 12:**
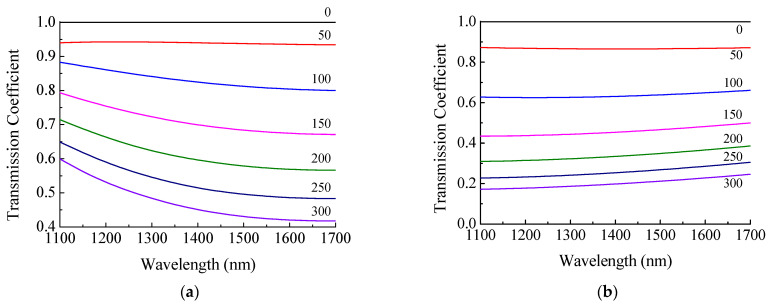
Transmission coefficients of (**a**) SM450 and (**b**) coreless fiber insertions versus wavelength for several insertion lengths.

The transmission of the insertion structure is a function of wavelength. These dependences for insertions without tapering of two types are shown in Figure 12. It can be seen that transmission of the SM450 fiber insertion is more dependent on wavelength than the transmission of the coreless fiber insertion. This can be explained as follows. At operating wavelengths (488–633 nm), the fundamental mode of SM450 fiber propagates through the core. At longer wavelengths, the fundamental mode cannot be confined in the core and goes through the cladding. When light enters the insertion as a core mode of SMF-28, it is decomposed into the modes of SM450. At shorter wavelength, the modes of two fibers have more similar field profiles at the input and less power is lost at the output. This leads to bending of the curves. The more we move away from the operating wavelength of this fiber, the lower the transmission of the fiber structure observed. The transmission of the structure based on the coreless fiber is practically independent of the wavelength, which is due to a weak dependence of the characteristics of cladding modes on wavelength in the absence of a core.

## 6. Experiment

### 6.1. Corning SMF-28 Fiber

**Figure 13:**
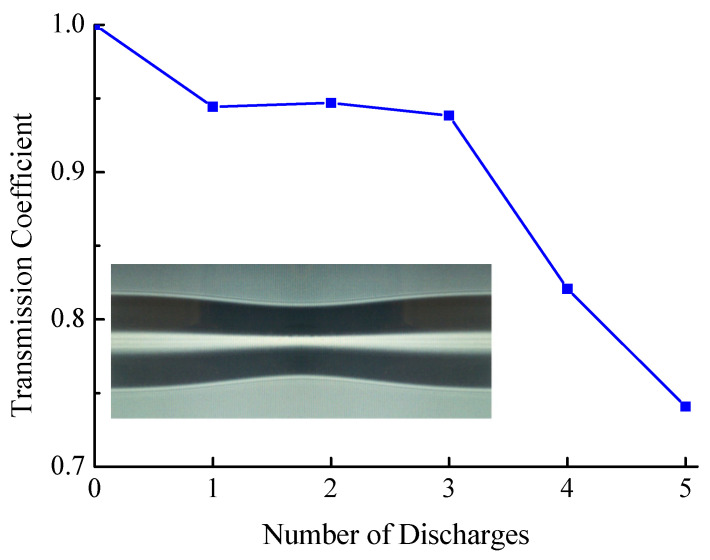
Dependence of transmission through tapered section of SMF-28 fiber. Inset shows a photo of the taper.

In order to verify the possibility of using the adiabatic approximation in calculating the transmission of tapered fiber structure, an experiment was first carried out to measure the transmission of standard fiber without insertion. In the adiabatic approximation, formation of a taper should not reduce transmission through the fiber because the modes in the taper smoothly transform into the corresponding cladding modes and back to the core mode. Figure 13 shows the change in transmission coefficient versus the number of discharges applied to the fiber without insertion. The discharge time was 220 ms. The transmission dropped from 1 to 0.75 after the fifth discharge, at which the taper diameter was 20 μm.

### 6.2. Fibercore SM450 Fiber

**Figure 14:**
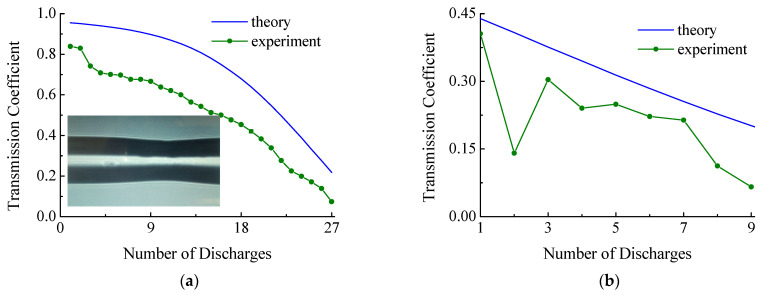
Transmission of the SM450 insertion with initial lengths of (**a**) 40 µm and (**b**) 300 µm during tapering. The inset shows a photo of the taper made from the screen of the splicing machine at the beginning of the process.

To confirm the theoretical results, two experiments were performed with SM450 fiber. In the first experiment, a structure was created with a 40-μm-long SM450 fiber insertion. Twenty-five discharges, each t=190 ms, were applied to the fiber insertion. In this case, the transmission decreased from 0.85 to 0.1 (Figure 14a). Based on the results of previous calibration measurements of the dependence of taper radius on the number of discharges (Figure 3b), the theoretical transmission curve of the structure was plotted in Figure 14a.

In the second experiment, a structure was created with a 300-μm-long SM450 fiber insertion. Nine discharges, each t=200 ms, were applied to the fiber insertion. In this case, the transmission decreased from 0.4 to 0.07 (Figure 14b). The final radius of the taper was 38 microns. The figure shows a theoretical curve for comparison. 

Figure 14 demonstrates that, in general, the characters of the obtained theoretical and experimental curves are similar. This indicates that the theoretical model is consistent with the real experiment. A 15–20% difference between the theoretical and experimental curves may arise due to some additional insertion loss, such as splice loss and non-adiabaticity of the taper.

### 6.3. Coreless Fiber

**Figure 15:**
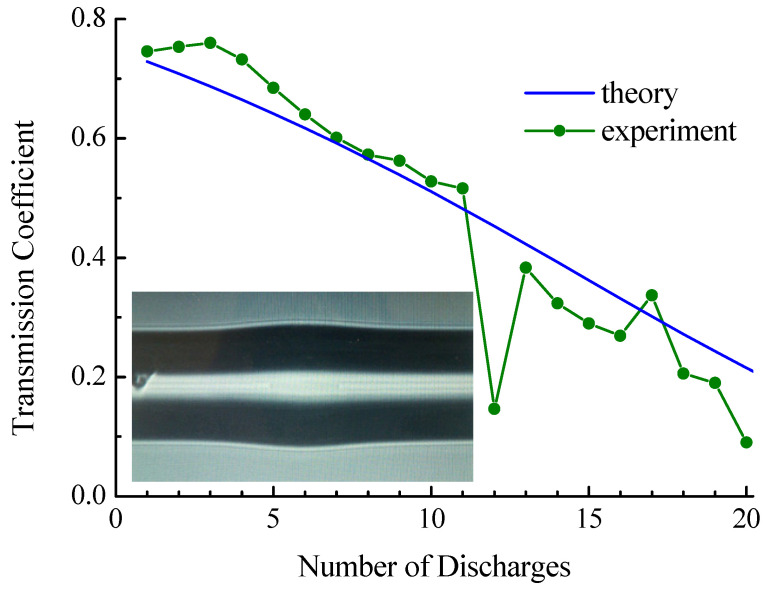
Transmission of coreless insertion with initial length 80 µm when it is tapered. The inset shows a photo of the taper.

In the experiment with the coreless fiber, the initial length of insertion was 80 μm. A photograph of the insertion in the initial state made from the screen of the splicing machine is shown in the inset in Figure 15. The cladding diameter of the coreless fiber was 140 µm, so some thickening is observed in the center of the picture made before tapering.

Figure 15 shows the dependence of the transmission on the number of discharges. The transmission coefficient drops from 0.72 to 0.21. For coreless fiber, the initial radius is 70 μm. Calibration measurements of the dependence of taper radius on the number of discharges were made for a long peace of fiber (Figure 3b). However, when we made an insertion from this fiber, it shrank during fiber splicing and its radius became closer to the radius of the standard fiber. Therefore, the results of calibration cannot be directly used for calculation. In the experiment presented in Figure 15, we observed that the fiber radius decreased to 38 μm after 20 discharges for arc duration t=190 ms. To carry out the numerical calculation shown in the figure, we assumed that the dependence of fiber radius on the number of discharges follows a curve similar to SMF-28 (Figure 3b) with corresponding scaling.

There is deep dropping of the transmission at 12 discharges; the reason for this is not clear. The behavior of transmission coefficient for the number of discharges from 13 to 20 is somewhat lower than it is before the drop. The correspondence between the theoretical curve and the experimental data shows that the splicing was well performed and that no radiation losses at the fiber splices are observed in contrast to the case of SM450 fiber. We are not able to control the initial length of insertion fabricated by cleaving with accuracy better than tens of micrometers. Therefore, it is hardly possible to check the reproducibility of the experimental results presented in Figure 13, Figure 14 and Figure 15.

## 7. Conclusions

We demonstrated that the tapering of fiber insertion is a simple and convenient way to adjust the transmission of the fiber structure designed to create a fiber interferometer. We showed the possibility of a step-by-step increase in taper length and a decrease in its radius by applying successive electric arc discharges of a splicing machine to the fiber insertion. The preferred discharge time is about 200 ms for a pulling force 0.05 N and can be adjusted to obtain the desired change in taper length per discharge. For this discharge time, the required coupling to cladding modes is achieved by applying 5–15 discharges. An expression is obtained that relates the length of the taper and its radius. When the discharge time is more than 300 ms, the fiber breaks.

All the symmetric LP_0*i*_ modes of SMF-28, SM450, and coreless fibers are calculated. The total number of these modes is 80–120 in wavelength range 1100–1700 nm. The coupling coefficients between the core mode of the standard fiber and the modes of the SM450 and coreless fibers are found. It is shown that, in both cases, more than half of the energy propagates in the first five excited modes. For coreless fiber, the maximum coupling coefficient is observed for the LP_04_ mode.

Using the adiabatic approximation, the distributions of the electric field amplitude in the SM450 fiber insertion and in the coreless fiber as well as the dependences of the transmission on the insertion length, taper elongation, and wavelength were obtained. The transmission of the structure based on the coreless fiber is almost independent of wavelength, which is due to the weak dependence of the characteristics of cladding modes on wavelength in the absence of a core.

Good agreement between the experimental and theoretical results is demonstrated. The numerical simulation presented in this work can be used to find the optimal parameters of tapered insertion for obtaining the required coupling to the core and cladding modes. The proposed method of adjusting mode coupling in fiber by increasing the length of the insertion heated and stretched in electric arc discharges is very important for the fabrication of fiber sensors yielding enhanced sensitivity to the surface refractive index, which can be employed in chemical and biological sensing applications.

## Data Availability

The data presented in this study are available on request from the corresponding author.
